# Occurrence of Recombinant Canine Coronavirus, Picodicistrovirus, and Circovirus in Red Foxes (*Vulpes vulpes*) Implies Frequent Virus Transmission Events Among Carnivores

**DOI:** 10.1155/tbed/6681119

**Published:** 2025-06-04

**Authors:** Enikő Fehér, Gábor Kemenesi, Tamás Görföl, Yasmine Wazzani, Kornélia Bodó, József Lanszki, Dóra Máté, Eszter Kaszab, Marianna Domán, Zoltán Zádori, Zsófia Lanszki

**Affiliations:** ^1^Department of Microbiology and Infectious Diseases, University of Veterinary Medicine Budapest, Budapest, Hungary; ^2^National Laboratory for Infectious Animal Diseases, Antimicrobial Resistance, Veterinary Public Health and Food Chain Safety, Budapest, Hungary; ^3^National Laboratory of Virology, Szentágothai Research Centre, University of Pécs, Pécs, Hungary; ^4^Institute of Biology, Faculty of Sciences, University of Pécs, Pécs, Hungary; ^5^HUN-REN Balaton Limnological Research Institute, Tihany, Hungary; ^6^One Health Institute, Faculty of Health Sciences, University of Debrecen, Debrecen, Hungary; ^7^HUN-REN Veterinary Medical Research Institute, Budapest, Hungary

**Keywords:** canine coronavirus, carnivore, circovirus, mustelid, picodicistrovirus, recombination, red fox, vulpes

## Abstract

Although their pathogenicity is most often unclear, some canine viruses have been found to infect carnivores other than dogs. This study relies on the surveillance of coronaviruses in 206 saliva and fecal samples of huntable, sympatric canid and mustelid species captured in Hungary, such as the native red fox (*Vulpes vulpes*), European badger (*Meles meles*), golden jackal (*Canis aureus*), and stone marten (*Martes foina*), as well as the recently settled alien raccoon dog (*Nyctereutes procyonoides*). Metagenomics-based and direct sequence analysis were deployed to determine the genome sequence of coronaviruses identified in two specimens collected from red foxes. Near-complete genome sequences of two canine coronaviruses (CCoVs) were obtained, together with the complete genome sequence of a canine circovirus (CanineCV) and the near-complete genome sequence of a canine picodicistrovirus (CPDV) from one of the samples. These provided the first fox origin CCoV and CPDV sequence data, and the first recorded appearance of the CPDV in Europe. The results suggested that recombination is of great importance in the evolution of CCoV, CanineCV, and CPDV infecting dogs and wild-living carnivores, including the red fox and golden jackal. These are widespread in Central and Southeast Europe, and have large ranges, facilitating transmission of the multihost canine pathogens.

## 1. Introduction

The red fox (*Vulpes vulpes*) could be considered one of the most successful medium-sized carnivorous mammals, appearing in numerous subspecies and color variations in Europe, Asia, North America, Africa, and Australia. The secret of its success is that these are well adapted to both wild and human habitats, from forests to deserts, and from agricultural to urban areas [1, 2]. In Central Europe, red foxes are frequent visitors of settlements, thus often come into contact with domestic animals and humans, mostly in an indirect way [3–5]. On the other hand, people often hike with their pets, which also gives the chance to meet wild animals contaminated environment. Although their ranges may differ, other canids and mustelids are encountering humans, and more or less accustomed themselves to the human presence and residential areas. Together, these all increase the potential for transmission of microorganisms among the aforementioned hosts [2, 6].

Some pathogenic canine viruses occur in dogs, red foxes, and other carnivores as well, such as the parvoviruses, canine coronaviruses (CCoVs), canine distemper virus, adenoviruses, picornaviruses, or canine circovirus (CanineCV). In pets, these have a direct pathogenic role, but simultaneous infection could provoke more severe diseases. Less is known about the infections in wild carnivores [7–20].

CCoVs can be characterized by dynamic evolution. The positive sense, single-stranded RNA genome of the coronaviruses consists of two long open reading frames (ORFs) for the nonstructural proteins (ORFs, 1a, and 1b), and four ORFs for the main structural proteins, the spike (S), envelope (E), membrane (M) and nucleocapsid (N). The existence and number of the ORFs of accessory proteins depend on the virus species or even the strain. The much-touted S protein is responsible for the viral cell attachment and entry, and its high mutation and recombination ability shape the properties of the virus and the course of infection [9, 21].

CCoVs may have a role in the development of mild-to-severe enteritis of dogs depending on the genetic characteristics of the strain, and the co-infective pathogens, but certain representatives have also been associated with fatal, systemic diseases [8, 9, 18, 22–25]. CCoVs belong to the *Alphacoronavirus* genus of the *Coronaviridae* family together with the feline coronaviruses (FeCoVs) and the transmissible gastroenteritis virus (TGEV). Type-I CCoVs (CCoV-I) and type-I FeCoV (FeCoV-I) might have evolved from a common ancestor, while CCoV-II, FeCoV-II, and TGEV represent a distinct evolutionary lineage with a history of multiple recombination among each other and often unclear parental network [9, 16, 21, 23, 26, 27]. As a major structural difference, CCoV-I has a cleavage site in the S, as well as an additional open reading frame (ORF3) basically missing from the CCoV-II genomes. However, the emergence of CCoV-I and -II recombinants makes the world of CCoVs even more interesting. CCoV-II subtypes (e.g., CCoV-IIa, -IIb, and -IIc) are classified based on the N-terminal domain (NTD, the 5' part of the S1 region) of the S, a particular site of recombination [9, 16, 21, 23, 26, 27].

In terms of the genomic structure, an interesting picornavirus-like virus, called canine picodicistrovirus (CPDV, previously named cadicivirus A; species *Dicipivirus acadici*, *Dicipivirus* genus, *Picornaviridae* family) has been described in dogs in China, 2012 [28]. Although the genome organization resembled that of picornaviruses, the novel genome exhibited two internal ribosome entry site (IRES) elements and two main ORFs, thus dicistronic organization with a layout of 5-UTR^IRES-II^-[1A-1B-1C-1D]-IGR^IRES-I^[2A-2B-2C/3A-3B-3C-3D]-3-UTR-poly (A) [28]. The pathogenic role of CPDV is obscured, and few sequence data are available from metagenomics studies. CPDV sequences have also been found in carnivores other than dogs, including fecal samples of raccoon dogs in China, and in red fox feces collected in rural and urban areas in Australia; unfortunately, in the latter case, only partial sequences have been submitted to the GenBank [10, 29].

Although some members could have a direct pathogenic role, circoviruses (*Circovirus* genus, *Circoviridae* family) are notorious because of their immunosuppressive effect [30–32]. Fox circovirus was first described in the serum and brain specimens of red foxes with neurologic signs [33]. Sequence analysis revealed an unequivocal relationship between the fox circovirus and CanineCV, thus, these viruses have been classified into the same taxonomical group (*Circovirus canine*). These viruses are widely distributed, appearing in healthy and diseased carnivores, canids, and mustelids as well [11, 20, 31, 34].

Here, as part of a scouting study, saliva and rectal samples of huntable canids and mustelids were processed for coronavirus surveillance to explore the virus diversity in the sampled geographical area. Metagenomics analysis was carried out on samples that tested positive, and, in addition to the CCoV, CPDV and CanineCV sequences were found and investigated in-depth from one specimen. The results suggested that recombination may have a major role in the evolution of virus strains obtained from wild living carnivores.

## 2. Materials and Methods

### 2.1. Sample Collection

The investigated saliva and rectal swab samples originated from 206 huntable animals, including red fox (*n* = 165), European badger (*Meles meles*, *n* = 24), golden jackal (*Canis aureus*, *n* = 13), stone marten (*Martes foina*, *n* = 3) and raccoon dog (*Nyctereutes procyonoides*, *n* = 1). The samples were collected by professional hunters in Central Europe, in Northeastern Hungary (Borsod-Abaúj-Zemplén county, Taktaköz) between December 2021 and March 2022. The saliva and rectal swabs were placed in the same tube natively and immediately stored at −20°C until delivery to the National Laboratory of Virology, University of Pécs, Pécs, Hungary, where they were processed.

### 2.2. Nucleic Acid Extraction and PCR

The sample swabs were washed in 400 μL phosphate-buffered saline (PBS) with extensive vortexing. Total RNA was extracted using Direct-Zol RNA MiniPrep Kit (Zymo Research, USA), and, following reverse transcription, the samples were screened with universal nested PCR primers for coronaviruses [35]. The first PCR of the nested system was carried out with the Luna Universal Probe One-Step RT-qPCR Kit (New England Biolabs, Ipswich, MA, USA). The PCR mixture of 20 μL contained 10 μL 2x Luna Universal Probe One-Step Reaction Mix, 3 μL nuclease-free water, 1 μL Luna WarmStart RT Enzyme Mix, 0.5 μL of each primer mix (200 nM of equimolar mixture: PC2S2−1, PC2S2−2, and 900 nM of equimolar mixture: PC2AS1-1, PC2AS1-2, PC2AS1-3), and 5 μL of the nucleic acid. The cycling protocol consisted of the steps: reverse transcription at 55°C for 10 min; denaturation at 95°C for 1 min; a touchdown protocol with 10 cycles, with the steps denaturation at 94°C for 20 s, annealing starting at 62°C with a decrease of 1°C per cycle for 30 s, and extension at 60°C for 40 s; 30 cycles with the steps denaturation at 94°C for 20 s, annealing at 52°C for 30 s, and extension at 60°C for 40 s; a final extension step at 60°C for 5 min. The second PCR was executed using GoTaq G2 Flexi DNA Polymerase Kit (Promega, WI, USA). In this case the PCR mixture of 25 μL contained 5 μL 5x Green GoTaq Flexi Reaction Buffer, 13.75 μL Nuclease-free Water, 0.25 μL GoTaq G2 Flexi DNA Polymerase (5 U/µL), 1 μM dNTP mix, 2 μL MgCl2 Solution (2.5 mM), 0.5 μL of each primer (80 nM of equimolar mixture: PCS-1, PCS-2, and 400 nM of primer: PCNAS), and 2 μL of the nucleic acid. The cycling protocol consisted of the steps: denaturation at 94°C for 2 min; 30 amplification cycles of denaturation at 94°C for 20 s, annealing at 60°C for 30 s, and extension at 72°C for 30 min; a final extension step at 72°C for 5 min. The PCR products were analyzed by standard agarose gel electrophoresis.

The sequence gaps, remained after assembling the reads generated with next-generation sequencing (NGS) were completed by PCR. Oligonucleotides were designed based on the contigs and used for amplification with DreamTaq DNA polymerase (Thermo Fisher Scientific, Waltham, MA, USA) according to the manufacturer's instructions (data not shown). The oligos were used for direct sequencing, executed by service companies.

### 2.3. Enrichment of Viral Sequences

Before NGS, an enrichment step was applied to increase the proportion of the viral origin reads [36]. The original samples containing coronavirus sequences were centrifuged at 16,000 × *g* for 10 min, and 160 μL of each supernatant was filtered through a centrifuge membrane filter with pore size of 0.45 μm (Ultrafree-CL Centrifugal Filter, Merck Millipore, Darmstadt, Germany). One hundred and fifty μL of the filtered samples were treated with a cocktail of 1 μL micrococcal nuclease (New England Biolabs, Ipswich, MA, USA), 2 μL of benzonase (Merck Millipore, Darmstadt, Germany), 4.5 µL of Turbo DNase, and 15.5 µL Turbo DNase Buffer (Thermo Fisher Scientific, Waltham, MA, USA) for 2 hours at 37°C, and were extracted with the Direct-zol RNA MiniPrep Kit (Zymo Research, USA).

### 2.4. NGS

RNA library was generated using the NEBNext Ultra II Directional RNA Library Prep Kit for Illumina (New England Biolabs, Ipswich, MA, USA). Briefly, 10 ng of the RNA was used as input for fragmentation and for cDNA generation using random primers. Thereafter, the cDNA was end-prepped and adapter-ligated, and then the libraries were amplified according to the manufacturer's instructions. The quality of the libraries was checked on an Agilent 4200 TapeStation System using D1000 Screen Tape (Agilent Technologies, Palo Alto, CA, USA), while the quantity was measured on Qubit 3.0 Fluorometer (Thermo Fisher Scientific, Waltham, MA, USA). Illumina sequencing was performed on a NovaSeq 6000 instrument (Illumina, San Diego, CA, USA) in a 2 × 151 cycles run configuration.

### 2.5. Bioinformatics

Illumina reads were *de novo* assembled with MEGAHIT v.1.2.9. The contigs were checked with the basic local alignment search tool (BLAST) against the NCBI GenBank database. The closest related sequences were used as references for mapping of the raw Illumina reads with the built-in mapper of Geneious Prime v.2024.0.3. The resulting sequences were checked manually to reveal possible errors. Raw reads were remapped to the resulting genomes for another manual check. As indicated above, the sequence gaps were filled with reads generated by direct sequencing. The genomes were annotated with Geneious Prime v.2024.0.3 and the Open Reading Frame Finder (https://www.ncbi.nlm.nih.gov/orffinder/).

The Kraken2 v1.0 (database PlusPF) was used for the taxonomic classification of raw reads, and the results were visualized with KronaTools and Pavian [37–39].

The generated genomic sequences were edited and aligned with the MUSCLE algorithm of the AliView software, or the MAFFT algorithm of the Geneious Prime software [40]. Pairwise identity values and neighbor-joining phylogenetic trees were generated with the MEGA 11 software [41]. Maximum likelihood phylogeny was investigated with the PhyML 3.0 online tool [42]. Recombination analysis was carried out with the Recombination Detection Program 4, using the RDP, GENECONV, BootScan, MaxChi, Chimera, SiScan, and 3Seq methods [43].

## 3. Results

### 3.1. PCR and Metagenomics

Using the diagnostic nested PCR system, coronavirus sequences were detected in two red fox samples of a juvenile female (shot on 22.01.2022) and a juvenile male (shot on 14.02.2022) [35]. Both samples were further investigated with metagenomics tools.

**Figure 1:**
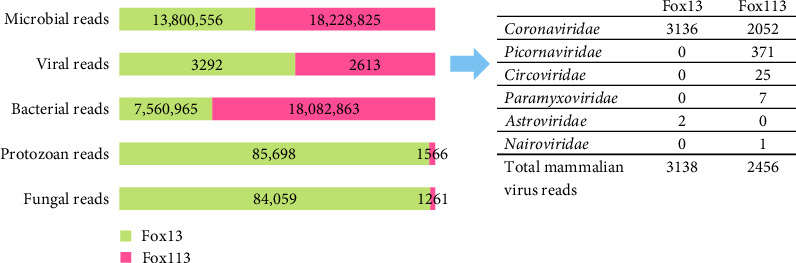
Results of metagenomics sequencing prepared from two red fox samples (fox13 and fox113), representing the classified read numbers.

Altogether 13,800,556 and 18,228,825 microbial reads could be identified from the processed sample fox13 and fox113, respectively. Despite the filtration and DNase/RNase digestion, the number of reads matching viruses was relatively low. According to the Kraken2 program, 3138 reads derived from sample fox13, and 2456 reads obtained from sample fox113 mapped to mammalian virus sequences, mostly to coronaviruses (*Coronaviridae*), as well as to picornaviruses (*Picornaviridae*) and circoviruses (*Circoviridae*) in the case of the sample fox113 (Figure 1). The number of bacteriophage sequences was relatively low (4.7% and 6% for the samples fox 13 and 113, respectively).

### 3.2. Coronaviruses

One long, *de novo* assembled sequence contig, generated from both red fox samples, matched with CCoV sequences. Altogether, 6459 (average depth of 34) and 4317 reads (average depth of 23) mapped to the CCoV references (chosen by BLAST analyses) in the case of the sample fox13 and sample fox113, respectively. Completion of the sequence gaps with direct sequencing resulted in a 28,855 (sample fox13) and a 28,955 (sample fox113) nt long, near-complete genome sequence that showed 95.1% genome-wide identity with each other. The sequence of the CCoV strain fox13 represented ≤94.3% overall nt identities with that of the closest related references, CCoV/GD/2020/X9 (GenBank acc. no. MZ320954, from a Chinese bamboo rat or *Rhizomys sinensis*, China, 2020), 61/22 (GenBank acc. no. OX335623, from a dog, UK, 2022), and CB/05 (GenBank acc. no. KP981644, from a dog, Italy, 2005). In case of CCoV strain fox113, this value was ≤94.1%, if compared to the sequence of the reference strains 11/22 (GenBank acc. no. OX335541, from a dog, UK, 2022), 61/22, and CCoV/GD/2020/X9. The 5' and 3' ends of both the CCoV fox13 and CCoV fox113 genomes were incomplete, with missing a 6 nt and an 84 nt long overhang, respectively, as revealed by comparisons to CCoV complete genome sequences (strains CB/05 and 11/22).

**Figure 2:**
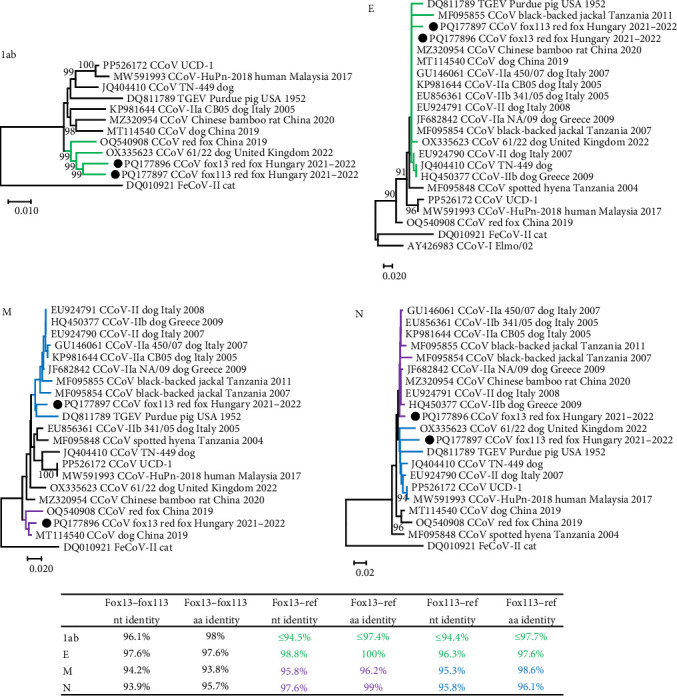
Unrooted, neighbor-joining phylogenetic tree of canine coronavirus 1 ab, nucleocapsid (N), envelope (E), and membrane (M) protein amino acid sequences, generated using p-distance model and 1000 bootstrap replicates. Branch support values <80 were hidden. The scale bar indicates substitutions per site. The novel strains are labeled with black circles. The table contains the highest pairwise identity values calculated by sequence comparisons. Green color shows the tree branches and data in the table, where the fox13 and fox113 located on a common branch. Purple and blue highlight represent branches and data connected to the fox13 or fox113 strains, respectively.

The structure of the genomic region 1ab of the CCoV fox13 and fox113 was comparable with that of the CCoV-II strains, including the closest related sequences of the strain 61/22 and VuCCoV_191134SY (GenBank acc. no. OQ540908, from a red fox, China, 2019). Considering the 1ab, the CCoV fox13 and fox113 represented 96.1% nt and 98% aa identity with each other, and ≤94.5% nt and ≤97.7% aa identity with the references (table in Figure 2).

**Figure 3:**
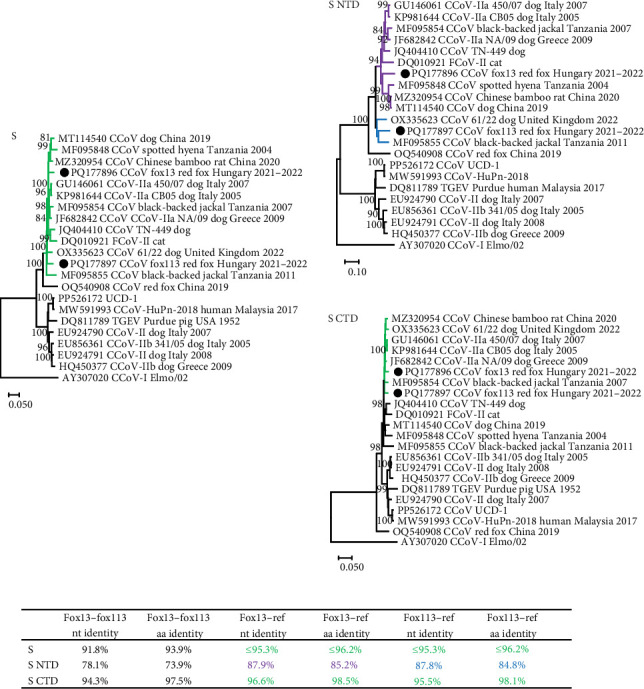
Unrooted, neighbor-joining phylogenetic tree of canine coronavirus spike protein (S) amino acid sequences, generated using p-distance model and 1000 bootstrap replicates. Branch support values <80 were hidden. The scale bar indicates substitutions per site. NTD: N-terminal domain of the S; CTD: C-terminal domain of the S, including the receptor binding region of the S1, and the S2 region. The novel strains are labeled with black circles. The table contains the highest pairwise identity values calculated by sequence comparisons. Green color shows the common tree branches and data concerning both fox13 and fox113. Purple and blue highlight represent branches and data connected to the fox13 or fox113 strains, respectively.

The NTD of the two novel CCoV strains (669 nt and 223 aa for the strain fox13, 672 nt and 224 aa for the strain fox113) showed moderate, 78.1% nt and 73.9% aa identity with each other. This part of the fox13 CCoV genome represented ≤87.9% nt and 85.2% aa identity with the closest relatives, sequences gained from a Chinese bamboo rat (CCoV/GD/2020/X9), a spotted hyena (*Crocuta Crocuta*; strain SH36_2004, GenBank acc. no. MF095848, Tanzania, 2004) and a dog (strain B447_ZJ_2019, GenBank acc. no. MT114540, China, 2019). This group of sequences clustered with the CCoV-IIa type strains CB/05 and 450/70 (GenBank acc. no. GU146061, from a dog, Italy, 2007) (Figure 3). The nt and aa pairwise identity values were ≤87.8% and 84.8%, respectively, for the NTD of the CCoV strain fox113 if compared to the reference CCoV sequences identified in the samples of a dog (61/22) and a black-backed jackal (*Canis mesomelas*; GenBank acc. no. MF095855, Tanzania, 2011). Comparison of the aa alignment revealed the absence of a sialic acid binding domain in the NTD of CCoV fox13, fox 113, and the reference sequences clustering with these [23, 27].

The remaining, 1229 aa long part of the S (downstream of the NTD, referred here as CTD) of the CCoV fox13 and fox113 genomes showed 94.3% nt and 97.5% aa pairwise identity with each other, and even higher pairwise identities with the closest references (table in Figure 3). The high identities and close clustering were maintained through the 3a, 3b, 3c, and E regions, and the CCoV-IIa and CCoV-IIb strains were located on the same branch of the phylogenetic tree (represented by the tree E, Figure 2). ORF3, typical for CCoV-I strains, could not be identified in the CCoV fox13 and fox113 genomes. The relationships of the CCoV fox13 and fox113 strains varied for the M and N. Regarding M, the fox13 CCoV strain clustered with the dog origin strain B447_ZJ_2019 and red fox origin strain VuCCoV_191134SY, while CCoV fox113 was located in a common branch with TGEV, IIa, IIb, and jackal origin strains. In contrast, in the N-based phylogenetic tree, the CCoV fox13 clustered with IIa, IIb, and jackal origin strains, while the fox113 branched with the TGEV, the dog origin 61/22, UCD-1, and human origin CCoV-HuPn-2018 strains (Figure 3). Overall, despite the variable grouping, the genetic distances were low between the novel strains in every region, with the exception of the NTD.

The putative, nonstructural 7a and 7b protein encoding genomic region represented low variability. However, some strains did not have any, or had truncated 7b that may be nonfunctional, or encode proteins with altered functions. A 180 nt and a 183 nt long deletion (interrupted with a short sequence that matches with references) were recorded in the middle part of the fox113 CCoV 7b genomic region, resulting in a 92 aa long protein, if it is functional at all. In contrast, a 459 nt long deletion shortened the 7b of the CCoV fox113, thus the derived protein might be only 60 aa in length. Shortened 7b regions with internal deletion have also been noted for other CCoV-II strains (for example, the sequence with GenBank acc. no. EU856361, from a dog, Italy, 2005).

**Figure 4:**
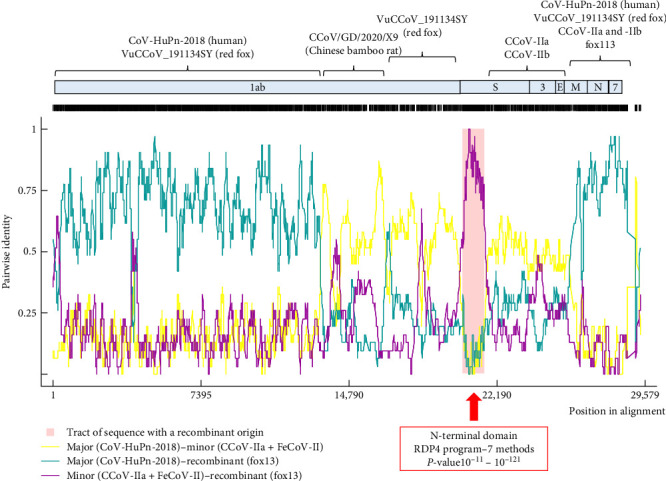
Representation of the recombination event affecting the N-terminal domain of the spike protein coding genomic region of the canine coronavirus strain fox13. The colored lines represent the pairwise relationships between the major parent, minor parent, and the recombinant. Other potential recombination events, the involved genomic regions and donor sequences are indicated above the plot.

Unsurprisingly, recombination analysis of coronavirus near-complete genomic sequences resulted in a number of recombination events. A high probability event listed the CCoV fox13 and fox113, and some dog origin strains (including the pantropic CCoV-IIa reference strain CB05, strain NA/09 with GenBank acc. no. JF682842, and strains 11/22 and 61/22) as recombinants (Figure 4). The affected genomic region spanned the NTD, with possible recombination points in the 3' end of the 1ab and in the receptor binding 5' region of CTD. The event implicated the human origin strain CCoV-HuPn-2018 (acc. no. MW591993) as major parent and FeCoV-II as minor. The clustering with the minor parent could be followed through the phylogeny as well; within the NTD and CTD tree, the FeCoV-II (GenBank acc. no. DQ010921) clustered close with the above-mentioned recombinants and IIa strains, but the difference became more apparent in the downstream and upstream genomic regions. The strain CCoV-HuPn-2018 was located on a separate branch of the NTD tree with the CCoV-IIb sequences. It should be noted that parents are difficult to unravel due to the multiple recombination, and the predicted relationships change for the variable genomic regions (Figure 4). Accordingly, CCoV-HuPn-2018, which has also been characterized as a recombinant strain, was phylogenetically localized closer or further to the fox13 and fox113, depending on the investigated genome fragment (Figure 4) [44]. Phylogeny and pairwise comparison suggested the red fox origin strain VuCCoV_191134SY and the dog origin strain 61/22 as the closest relatives of both the CCoV fox13 and fox113 for the 1ab, which changed variably in the downstream regions. The phylogenetic analysis (Figures 2 and 3) was applied to complete ORFs, thus, it did not faithfully reflect the (multiple) recombinations. Recombination was also predicted within the 1ab of the fox13 and fox113 that was more pronounced in the second part of this ORF, affecting an approximately 5000 nt long fragment potentially obtained from the Chinese bamboo rat origin strain CCoV/GD/2020/X9 (Figure 4) [45]. Regarding the region downstream of the S to the 3' end of the M, IIa and IIb strains might serve as minors for the novel genomes. Then, as the phylogenetic trees also showed, the two novel strains branched somewhat separately, and the probable genetic donors of this region varied, also depending on the sequences included in the analysis; clear relationships could not be established.

### 3.3. Picodicistrovirus

**Figure 5:**
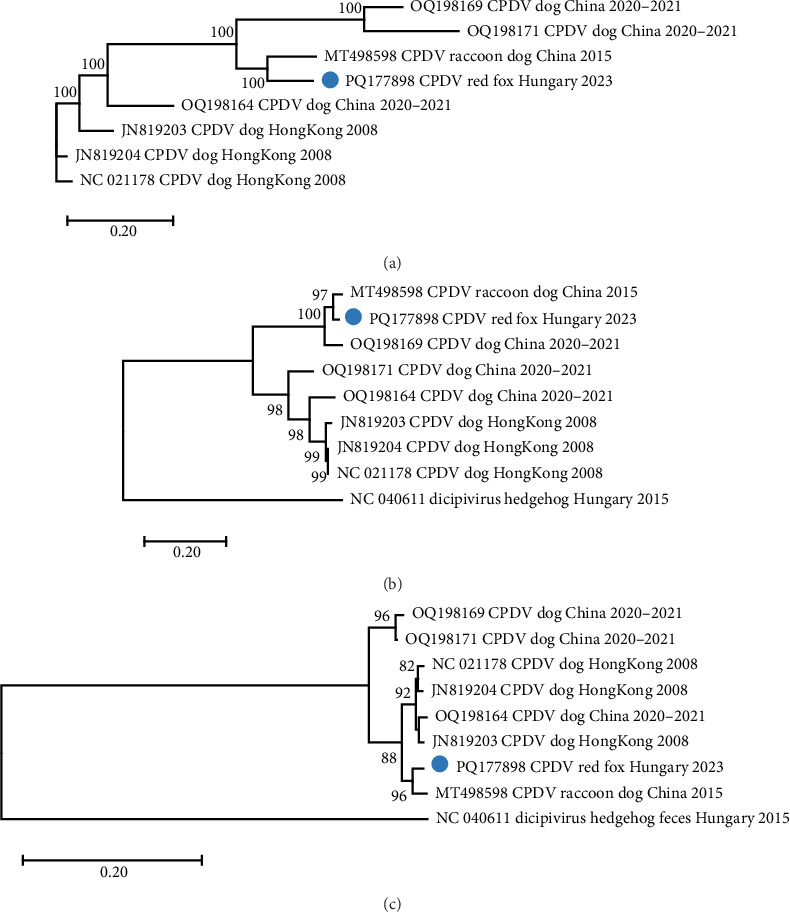
Maximum likelihood phylogenetic tree of canine picodicistrovirus near-complete genome nt sequences (panel A, unrooted tree generated with GTR + G model), as well as polyprotein 1 and polyprotein 2–3 amino acid sequences (panel B and C, respectively, generated with LG + R+F model, rooted to the hedgehog dicipivirus sequence). The trees were constructed using 1000 bootstrap replicates; branch support values <80 were hidden. The scale bar indicates substitutions per site. The novel strain was labeled with blue circle.

One contig, obtained by *de novo* assembly from the reads of the sample fox113, represented ≤87% nucleotide (nt) identity with CPDV strains according to BLAST search. The deeper bioinformatics analysis resulted in compilation of an 8793 nt long, near-complete genome sequence (mapping 1759 reads to it, average depth of 30) that showed the highest overall pairwise identity of 87.5% with a CPDV sequence identified in the feces of a common raccoon dog sampled in China, 2015 (strain RDX, GenBank acc. no. MT498598). The dicistronic organization of the novel sequence, together with the phylogenetic analyses, suggested that the generated sequence indeed derived from CPDV [28] (Figure 5).

**Figure 6:**
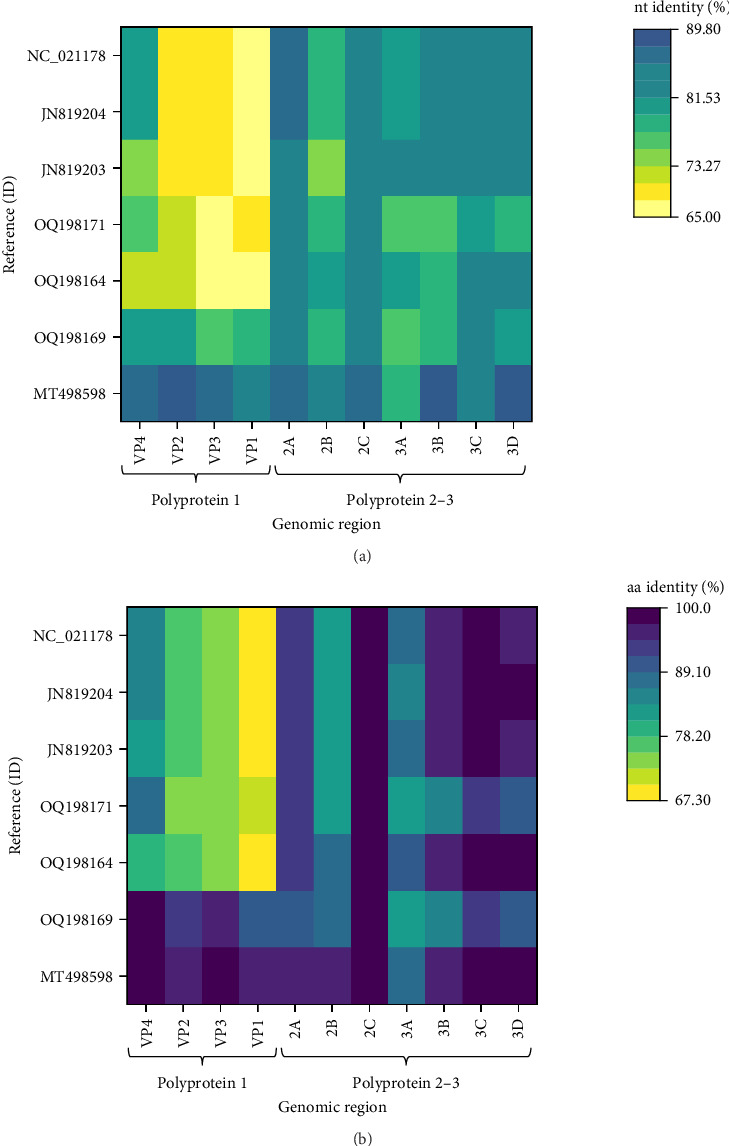
Representation of the nucleotide (A) and the amino acid (B) pairwise identity values calculated for the protein coding regions in the genome of the novel picodicistrovirus (GenBank acc. no. PQ177898) and reference sequences. The investigated genomic region and the GenBank accession number of the references are listed at the *X* axis and *Y* axis, respectively.

Two main ORFs were predicted in the novel genome with the layout typical for the CPDVs [28]. The viral capsid polyprotein (P1) sequence showed 86.6% nt and 96.8% aa identity with that of the strain RDX, while the nt and aa identities were 66.8%–78.5% and 72.2%–93.3%, respectively, with the P1 sequence of other reference CPDV strains (Table 1, Figure 6). The inferred P1 could be cut for four proteins, but a notable difference is in the length of the VP4 of the novel sequence and the available reference sequences (44 aa) compared to the VP4 of RDX (81 aa) [29]. The nt, but not the aa pairwise identities showed significant variances both for P1, and for the other polyprotein, the P2-3 (Table 1): the nt identities remained below 90% for all of the predicted proteins, while the aa identity is ≥95%, except the 3A of the P2-3 (89.7%) (Table 1).

The intergenic region (IR) of the novel sequence, intervening the P1 and P2-3, is 2 nt longer (599 nt) than that of the strain RDX. The two sequences showed high, 95.3% nt identity with each other. The P2-3, which encodes the nonstructural proteins, represented 86.5% nt and 97.3% aa identity with that of the strain RDX, while the identity was 80.9%–83.9% at nt and 91.3%–95.9% at aa level with the P2-3 region of other CPDV strains (Table 1, Figure 6). The RNA-dependent RNA polymerase sequence, encoded in the 3D of the P2-3 region, represented the highest identity with that of the strain RDX (89.8% for nt and 98.3% for aa), as well as with two red fox origin partial CPDV sequences (85.6%–86.6% nt and 98.8%–98.9% aa identity) [10]. The complete genome sequence of the CPDVs identified in red fox in Australia (GenBank acc. no MT833876 and MT833880) has not been determined, thus, further comparisons cannot be executed. When considering the aa identities, 2C (containing NTP binding and helicase motifs) seemed to be a conserved region with uniformly high identities (≥97.4%) in all comparisons (Figure 6). Although the nt identities represented uniformity as well, these were below the value of the aa identities. The coloring in Figure 6 highlighted the overall, relatively large differences between the corresponding nt and aa identities.

In addition to the complete coding region, 924 nt of the 5' end and 401 nt of the 3' end of the fox113 genome were determined. Some dog origin reference sequences (GenBank acc. no. JN819202-JN819204) have a shorter 5' noncoding region of 875–878 nt in length, and a 428–429 nt long 3' region, if excluding the poly (A) tail. In contrast, the RDX genome has an even longer 5' noncoding region (1063 nt), while its 3' end has not been completely determined.

**Figure 7:**
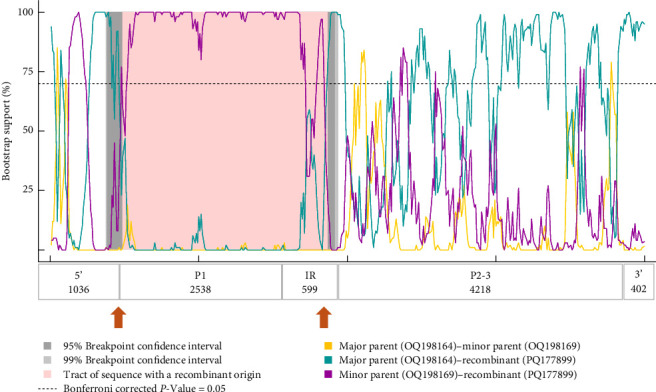
Schematic representation of the predicted recombination event affecting the genetic material of the canine picodicistrovirus sequence identified in red fox.

Recombination analysis of the available near-complete CPDV sequences indicated a probable recombination event among dog origin strains affecting almost the entire P1 (recombinant GenBank acc. no. OQ198171, major GenBank acc. no. OQ198169, minor GenBank acc. no. JN819203). Another event listed the novel CPDV fox113 as a recombinant, with dog origin sequences as the major and minor parents (Figure 7, predicted by 5 methods with *p*-values 10^−7^–10^−19^). The breakpoints were located at the very end of the 5' noncoding region and in the conserved, central part of the IR, thus, the recombinant region spanned the whole P1. The recombination can be followed through the clustering in the phylogenetic trees and the identity values in the matrix as well (Figures 5 and 6).

### 3.4. CanineCV

Some short contigs, assembled from the sequence reads obtained from sample fox113, matched with fox circovirus (referred to thereafter as CanineCV) sequences. Remapping to the closest related sequence (strain 55590, GenBank acc. no. KP941114, from fecal sample of a red fox, Croatia, 2014) resulted in the determination of three genomic regions built from 124 reads [31]. Overlapping PCR amplicons were produced and directly sequenced to complement and confirm the metagenomics results. The length and the structure of the novel complete genome (GenBank acc. no. PQ177899) were comparable with those of the other CanineCVs. The 2056 nt long genome contained two main ORFs, one for the *rep* (912 nt) and one for the *cp* (813 nt), as well as a 3' IR (203 nt) and a 5' IR (128 nt). The typical nonanucleotide motif TAGTATTAC is located upstream of the *rep* in the 5' IR.

**Figure 8:**
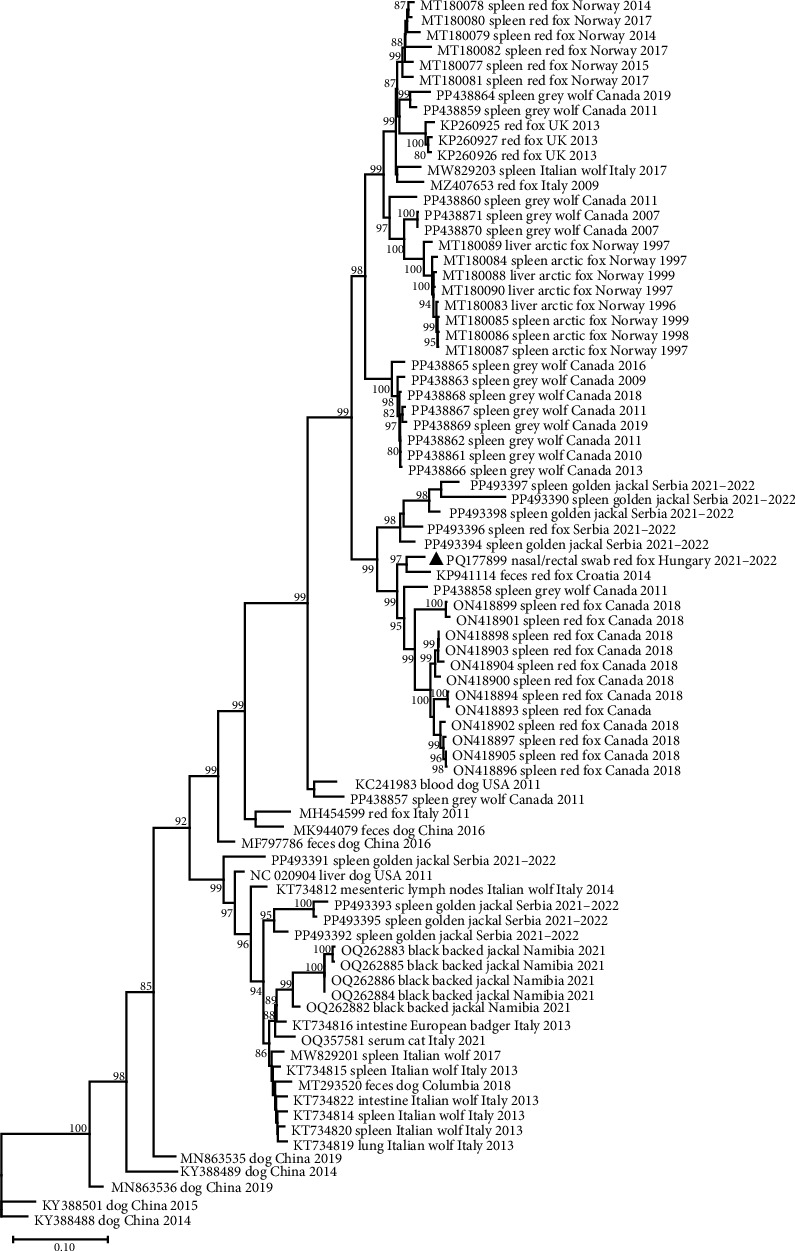
Unrooted, maximum likelihood phylogenetic tree of canine circovirus near-complete sequences, generated using GTR model and 1000 bootstrap replicates. Branch support values <80 were hidden. The scale bar indicates substitutions per site. The novel strain was labeled with a black triangle.

The genome sequence of CanineCV fox113 shared the highest nt identity of 96.2% with that of the strain 55590. These two sequences are located on a common branch in the phylogenetic tree generated using the near-complete genome sequences of the available CanineCV sequences of wild carnivores (e.g., red fox, arctic fox, jackals, and wolves) and some representative strains identified in dogs (Figure 8). Further closely related sequences have been detected in red fox in Serbia and Canada, in golden jackal in Serbia, as well as in gray wolf in Canada (Figure 8).

**Figure 9:**
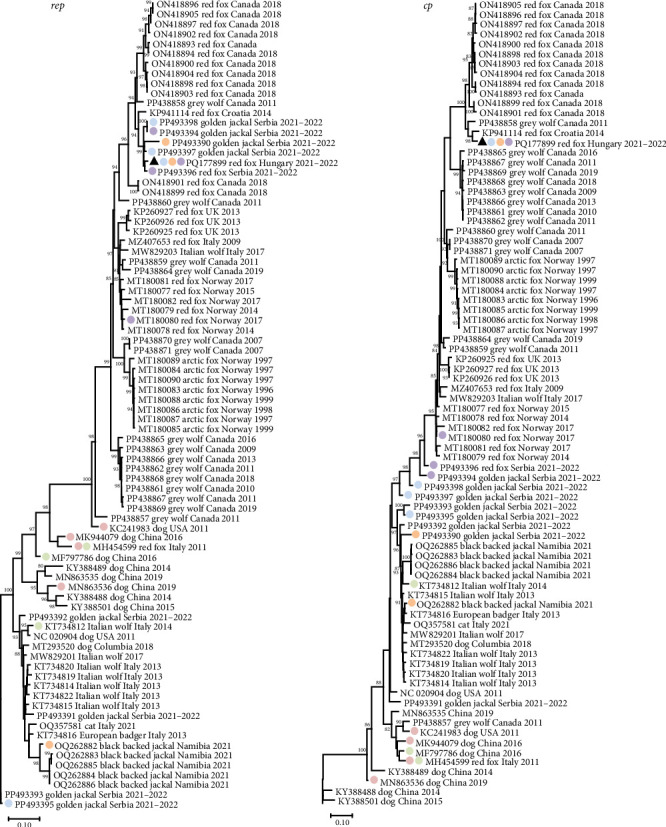
Unrooted, maximum likelihood phylogenetic tree of canine circovirus *rep* and *cp* nucleotide sequences, generated using GTR model and 1000 bootstrap replicates. Branch support values <80 were hidden. The scale bar indicates substitutions per site. The novel strain was labeled with black triangle. Colored circles indicated strains involved in recombination events containing fox origin sequences; identical colors refer to strains of a given event in Figure 10.

The CanineCV strain 55590 was among the closest relatives of the CanineCV fox113 in any comparisons, but the *rep* and *cp* (nt and aa) based phylogenetic trees represented differences otherwise. Regarding the *rep* and the deduced protein sequences, the novel strain clustered with the same references as found in the whole genome-based tree. In contrast, the Serbian, red fox, and golden jackal origin strains are located in separate branches of the *cp*-based nt and aa trees (Figure 9).

**Figure 10:**
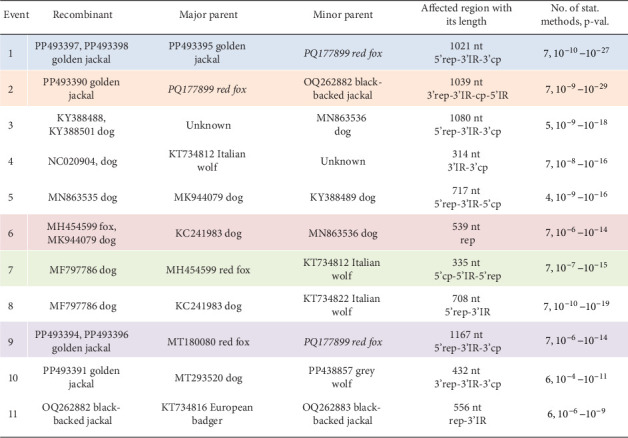
List of the probable recombination events predicted for canine circoviruses. The table contains the GenBank accession number of the strains, the host species, the affected genomic region, as well as the number of statistical methods supporting the recombination probability together with the calculated *p*-values. The novel strain is written in Italics. Events implicating fox origin canine circoviruses are highlighted, and the same coloring is used in the phylogenetic tree of Figure 9 to label strains involved in the recombination. rep: replication-associated protein encoding genomic region: intergenic region; cp: capsid protein encoding genomic region.

Recombination analysis was carried out to reveal the evolutionary processes accompanied by the exchange of genome fragments among wild carnivore origin CanineCVs. The novel red fox origin strain was involved in recombination events primarily as a major or minor parent. Potential recombination (event no. 1, Figure 10) occurred among CanineCV sequences identified in golden jackal from Serbia and the CanineCV fox113, affecting a 1021 nt long region overlapping most of the *rep*, the 3'IR, to the 3' part of the *cp*. Another recombination event (event no. 2, Figure 10) predicted the participation of the novel, red fox, and a black-backed jackal origin (from Namibia) sequences, resulting in a recombinant detected from golden jackal (from Serbia). The recombinant region was 1039 nt long, covering the complete 3' IR and *cp*. The CanineCV fox113 was found a minor parent in a third event affecting a 1167 nt long region containing a long part of the *rep* (event no. 9, Figure 10), in which case golden jackal origin sequences were identified as recombinants, while the major was a strain collected from a red fox, Norway. These three events included exchange of sequences overlapping most of the *rep* or *cp*, thus could be followed through the clustering in the ORF-based phylogeny (Figure 9, strains labeled with blue, orange, and purple circles according to Figure 10).

Additional recombination events were also statistically supported, implying sequences identified in red fox (also the sample fox113), golden jackal, black-backed jackal, Italian wolf, European badger, and dog. The exception was the arctic fox origin CanineCVs, which were grouped separately in each case, as described earlier, with two strains found in gray wolf specimens collected in Canada [46]. Recombination was previously suggested among red fox and dog origin strains, which was confirmed here [34, 47].

## 4. Discussion

Although the here studied sympatric (spatially and temporally coexisting) canid and mustelid species are of different social structures, each has a wide geographical distribution, considerable ecological plasticity, and the ability to resource partitioning [48–51]. The red fox, the stone marten, and the European badger are common species in Hungary, while the extinct and resettled golden jackal population increased in the country in recent decades [52, 53]. Expanding in Europe, the non-native, invasive raccoon dog colonized our country, thus could be occasionally captured in the studied area [54]. The invasive species cause changes in the ecosystems, modifying interspecific interactions, and may introduce novel pathogens as well. Along with this, the extent of the distribution area and the proximity of the hosts are usually associated with faster spread and emergence of microorganisms [2, 55–58].

Based on its high abundance and generalist habitat use, the red fox is most likely to come into contact with wild-living and domestic animals. Regarding the three viruses investigated in this study, the fox circovirus, classified as CanineCV, has been diagnosed in red fox and a wide variety of carnivores worldwide [20, 31, 33, 34, 46, 47, 59]. Although there were some separations between CanineCV of dogs and wild-living canids in the phylogeny, in some cases, closely related strains were collected from the two animal groups. Besides dogs, CCoV has also been found in foxes and other carnivores, but the phylogenetic clustering of these strains was less pronounced, probably due to the frequent recombination [60–62]. More sequence data from different geographic areas would be needed to reveal the factors responsible for the host specificity, tissue tropism, and virulence of CCoVs. CCoV sequences of red fox have been published from China and have been detected by PCR in Portugal in Europe [63–66]. Thus, sequence analyses of red fox origin CCoVs could be executed using the Chinese and Hungarian data. Very little is known about the third virus, the CPDV, which has not been reported in Europe so far. Although it has been identified in red fox samples in Australia, only partial genome sequences are available [10]. A few CPDV (near)-complete genome sequences, obtained from dogs and a raccoon dog, are available, but the results are suggestive of recombination. The appearance of the virus in three distant areas and three (supposed) host species raises the possibility of its wide distribution both geographically and in terms of host.

As mentioned, recombination plays a key role in the evolution of CCoVs and shapes the genome of CanineCVs as well [9, 16, 21, 23, 26, 27, 34, 47, 67]. As the phylogeny and recombination analysis reflected, the sequence relationships varied depending on the investigated genomic region, suggesting multiple exchanges of genomic fragments in the past among coronaviruses with diverse geographic and species origins. The genetic material of CCoV fox13 and fox113 might emerge through recombination of CCoVs of carnivores, or even rodents, that can serve as food for canids [45]. It is equivocal whether humans could be considered true hosts for CCoVs, and what factors might cause the CCoVs to infect humans [44, 68]. Thus, involvement of human-origin viruses in recombination affecting viruses of wild carnivores is questionable, but not inconceivable, indirectly through the dogs or another intermediate host (not forgetting cats and FeCoVs) by chance. People are living in ever-closer contact with their pets (particularly with dogs, cats, or ferrets, all interesting from the point of view of coronavirus infections), which could facilitate interspecies transmission of viruses, as we have seen for the severe acute respiratory syndrome coronavirus 2 (SARS-CoV-2) as well [69]. At last, it may lead to the adaptation of the microbes to a new host species. However, it is more likely that a person received CCoV that contained sequences closely related to the here-revealed recombinants.

Even if the strain fox13 and fox113 may be related to human-origin CCoVs, it does not necessarily mean increased zoonotic potential. The recombination was the most pronounced in the NTD region, in which case the CCoV fox13 and fox113 clustered in the phylogenetic tree with the CCoVs-IIa and FeCoV-II strains. These do not contain the motif evaluated as sialic acid binding domain, a possible virulence factor of the TGEV, and found in the NTD of the CCoV-IIb strains and the CCoV-HuPn-2018 [27]. CCoV fox13 and fox113 represented moderate pairwise identities and clustered with the NTD of pantropic CCoV-IIa strains causing systemic diseases in dogs [22]. However, a deletion in the 3b region, considered as a genetic marker of pantropic reference, could not be identified [9].

Recombination was predicted for the CanineCV sequences of wild carnivores. The phylogenetic analysis revealed a clear relation among the CanineCV strain fox113 and 55590, collected in Croatia from a red fox. The strains, detected from golden jackal in Serbia (sampled in 2021–2022 similarly to the CanineCV fox113) also represented high identities with these, but, along with the CanineCV fox113, were involved in recombination events in different contexts. The exchanges affected long genomic regions overlapping the complete, or most part of the *rep* or *cp* with the surrounding IRs, which meant a significant change for such a small genetic material with few coding ORFs. The golden jackal populations have been found to expand from the Balkans and the southern neighboring countries (including Serbia and Croatia) towards Northern and Western Europe, which favors (interspecies) transmission and recombination of variable CCoV strains [52, 70, 71]. The behavior of the hosts can exacerbate this, such as the large range size, the long dispersal distance, or a higher population density due to the alloparental care, typical for the golden jackal, or the red fox [49, 70, 72].

Although the here detected viruses may be of dietary origin, the relationships of the novel and reference strains raise the possibility that the foxes were indeed infected. Unfortunately, the mixed specimens we used do not allow us to infer the possible tropism and pathogenicity of the detected strains. A much more refined sampling, together with the description of the health status of the sampled animals, would be needed to gain more detailed, forward-looking results. It would be interesting to further investigate which species (including humans) could be hosts of these viruses in our country, and to reveal the genomic characteristics of the strains. Nevertheless, the circulation of the recombinant viruses draws attention to the problems caused by direct or indirect encounters of the wild carnivores and pets in an urban environment, or during outdoor leisure activities. The wide host spectrum of the three investigated viruses facilitates long-distance spread and recombination possibilities that increase the chance of a new pathogen emerging.

## 5. Conclusions

This study reports the detection and genomic characterization of CCoV, fox circovirus (classified as CanineCV), and CPDV strains identified in red foxes shot in Central Europe. In addition, these are the first CCoV sequences of red fox origin outside of China, and the very first CPDV sequences in Europe. It has been established that recombination is a major force in coronavirus genome evolution, but it also seems to be an important mechanism in the genomic diversification of CanineCVs and CPDVs, infecting both dogs and wild carnivores. The interspecies transmission and recombination could be followed through the CanineCV strains found in the specimens of the habitat generalist red fox and golden jackal in Hungary, and the neighboring countries, Croatia and Serbia.

## Figures and Tables

**Table 1:** List of the predicted genes and proteins in the genome of the canine picodicistrovirus identified in the fecal sample of a red fox in Hungary.

Region name	Region (nt)	nt length	Highest nt identity (%)	aa length	Highest aa identity (%)
*Polyprotein 1*	*1037–3574*	*2535*	*86.6*	*845*	*96.8*
1A (VP4)	1037–1168	132	86.4	44	100
2A (VP2)	1169–1879	711	89.2	237	96.2
3A (VP3)	1880–2629	750	86.1	250	99.2
4A (VP1)	2630–3571	942	85	314	94.9
*Intergenic region*	*3575–4173*	*599*	*95.3*	—	—
*Polyprotein 2–3*	*4174–8391*	*4215*	*86.5*	*1405*	*97.3*
2A	4174–4596	423	86.1	141	95
2B	4597–4971	375	83.5	125	95.2
2C	4972–5994	1023	86.3	341	99.4^a^
3A	5995–6255	261	81.6^b^	87	89.7^c^
3B	6256–6333	78	88.5	26	96.2
3C	6334–6939	606	85^c^	202	99.5
3D	6940–8388	1449	89.8	483	98.3

*Note:* The table represents the pairwise identity values calculated by comparison of the novel sequence to the relevant regions of the closest relative, the common raccoon dog picodicistrovirus (GenBank acc. no. MT498598), with exceptions indicated in the footnote. Italics emphasize the main parts of the genome with their relevant regions listed below.

^a^GenBank accession number JN819204.

^b^GenBank accession number JN819203.

^c^GenBank accession number OQ198164.

## Data Availability

The assembled genome sequences have been deposited in the GenBank database with accession numbers PQ177896–PQ177899.
